# Parents’ lived experience of living with and caring for their burn-injured child in a home setting

**DOI:** 10.1080/17482631.2023.2216032

**Published:** 2023-05-26

**Authors:** Lina Sophie Toft Lernevall, Asgjerd Litleré Moi, Eva Gjengedal, Pia Dreyer

**Affiliations:** a Department of Plastic, Hand and Reconstructive Surgery, National Burn Centre, Haukeland University Hospital, Bergen, Norway; b Department of Global Public Health and Primary Care, University of Bergen, Bergen, Norway; c Department of Health and Caring Sciences, Western Norway University of Applied Sciences, Bergen, Norway; d Department of Public Health, Section of Nursing, Aarhus University, Aarhus, Denmark

**Keywords:** Burns, home care, lived experience, needs, pediatric, parents, phenomenology, qualitative research, ricoeur, support

## Abstract

**Purpose:**

When a burn injured child is discharged from hospital to its home, the responsibility for the after-care treatment is transferred to the parent(s). A knowledge gap exists concerning how parents experience caring for a burn-injured child at home after discharge. The aim is to gain an in-depth understanding of parents’ lived experience of living with and caring for their burn-injured child in a home setting.

**Methods:**

Twenty-four parents of burn-injured children treated at a Norwegian burn centre were interviewed 74 to 195 days after the burn accident (June 2017 to November 2018). A phenomenological hermeneutic approach was chosen, using a Ricoeur-inspired textual in-depth analysis method. NVivo 12 Plus and COREQ were used.

**Results:**

Four themes emerged. The parents’ experienced feelings had been embodied and would stay forever. They felt left alone to continue the medical treatment at home without having the necessary skills. The parents grieved over the lost past and feared the unknown future. They longed to meet or be contacted by staff members who knew them and their life situation.

**Conclusions:**

Healthcare professionals should see returning home as part of the course of illness and that right support during the hospital can prevent challenges after discharge.

## 1. Introduction

Burn injuries are among the four most common unintentional injuries worldwide (Norton & Kobusingye, 2013). In Europe, children account for 40–50% of the severe burn injuries (Brusselaers et al., 2010). When a child is severely burn injured the whole family is affected (Bakker, Maertens, et al., 2013), and parents, in particular, are prone to impairments in mental health after their child’s accident (Enns et al., 2016). Paediatric burn injuries are often caused by hot liquids (scalds), hot solids (contact burns) and flames (flame burns) (Brusselaers et al., 2010). In many cases one or both parents were present at the time of the burn accident but unable to prevent it from happening (Pardo et al., 2008). The most common reactions in parents of a burn-injured child during the acute phase are anger (M. Egberts et al., 2017; McGarry et al., 2015), guilt, shame and blame (Hawkins et al., 2019; Kornhaber et al., 2018), which may develop into anxiety and depression (Hawkins et al., 2019).

During the long-term phase, parents are reported to suffer from self-doubt (Horridge et al., 2010) and posttraumatic stress (Bakker, Van Der Heijden, et al., 2013; De Young et al., 2014). Symptoms of anxiety, depression and posttraumatic stress, decline over time (Bakker, Van Der Heijden, et al., 2013; De Young et al., 2014; M. Egberts et al., 2017; M. R. Egberts, van de Schoot, et al., 2018). However, some parents will be psychologically burdened by their child’s burn injury many years after the burn accident (Bakker et al., 2010; Horridge et al., 2010; Sveen & Willebrand, 2018; Willebrand & Sveen, 2016). Parents’ psychological reactions during the acute stage following a burn accident are well documented as mentioned above. However, knowledge on parental experiences during in-hospital stay is sparse. Newer research has put focus on parental experiences during the inpatient stage such as parents’ experiences of their needs for support when having a child admitted to a burn centre (Lernevall et al., 2021) and parents’ experiences of their presence or absence during wound care (M. R. Egberts, de Jong, et al., 2018). For parents to positively contribute to their burn injured child’s treatment and recovery Lernevall et al. found four parental needs; being cared for as one whole family; feeling calm and safe in the hands of professionals, gaining some control over the situation; and getting breaks during the day to see to their own fundamental needs (Lernevall et al., 2021). Egberts et al. found that parents preferred being present during wound care, as it gave them some control to cope with their situation (M. R. Egberts, de Jong, et al., 2018).

Being discharged from a burn centre is filled with contradicting feelings and challenges for the parents. The after treatment of the healed burn wounds, transplanted skin or donor sites may require life-long rehabilitative and physical therapy to reduce scarring, contractures and support skin functionality (Mathias & Srinivas Murthy, 2017). Children in particular are exposed to these challenges while growing up (Celis et al., 2003). Rehabilitation and scar prevention therapy is expensive (Mirastschijski et al., 2013), and burn injury is associated with high personal costs for the child and its parents (Bakker, Maertens, et al., 2013). In addition, long-term rehabilitation involves social reintegration challenges and problems with body image and self-esteem (Hornsby et al., 2020). Earlier studies have indicated that parents long for their normal routines; however, as they now again have sole responsibility for their child, they also have fears that they will not be capable of continuing the prescribed treatment (McGarry et al., 2015; Ravindran et al., 2013a; Öster et al., 2014). Parents may also experience changes within themselves as they may become overprotective of their child and have difficulties sleeping (McGarry et al., 2015). On top of this, they have to deal with the long journey of recovery and public reactions to their child’s scars (McGarry et al., 2015; Ravindran et al., 2013a). The child’s outcome and wellbeing after a burn injury highly depend on the parents’ psychological reactions and on having a supportive and functional family (Bakker, Maertens, et al., 2013). Parents’ reactions and ways of coping, as well as how they are supported in dealing with their experiences, are therefore important for the recovery process of their burn injured child.

No model of care delivery exists for families of burn injured children after hospital discharge (Curtis et al., 2016), and a knowledge gap exists concerning parental experience in relation to life at home after their burn-injured child has been discharge. A more in-depth understanding of parents’ perspectives may facilitate tailored follow-up, which is essential for these parents not only in the immediate aftermath of the burn injury but also in a post-hospital perspective. Therefore, this study aims to gain an in-depth understanding of parents’ lived experience of living with and caring for their burn-injured child in a home setting.

## 2. Materials and methods

This study belongs to a larger study investigating the needs of parents during the course of hospitalization and follow-up of their child’s accidental burn injury (Lernevall et al., 2019, 2020, 2021). This qualitative study sought to understand the parents’ lived experiences and thereby their lifeworld, wherefore a phenomenological-hermeneutic methodology was chosen. Within phenomenology the researcher is interested in “meaning structures of the lived world” (Ricoeur, 1976), by looking, understanding and describing things as they show themselves.

One extensive description of phenomenology and hermeneutics has been offered by the French philosopher Paul Ricoeur (1913–2005). He argued that “the belongingness to the world is the interpretive experience itself and that all understanding is mediated by interpretation” (Morse, 1994, p. 121). The analysis method in the present study is therefore a Paul Ricoeur-inspired textual analysis method (P. S. Dreyer & Pedersen, 2009).

### 2.1. Participants and recruitment

Twenty-four parents participated in this study (12 mothers, 11 fathers and 1 stepfather) (Table I), of whom 21 parents had participated in an earlier interview (Lernevall et al., 2021). Inclusions criteria were parents to children <12 years, who were hospitalized minimum 24 hours at the burn centre due to an accidental burn injury (Lernevall et al., 2021). The guiding principle for the sample size was information-richness and variation to experiences, which provided a data material with in-depth descriptions and stories to elucidate our aim (Malterud, 2012). For the first interview, described in another article (Lernevall et al., 2021), 22 parents were recruited purposive by four psychologists. Within the first days after admission to the burn centre all parents were seen by a psychologist who invited the parents fulfilling the inclusions criteria to participate in the study. Thirty invitations were handed out.

**Table I.** Characteristics of sample.

Children	*N* = 12	Parents	*N* = 24
Girls/boys, n	4/8	Mothers/fathers/stepfather, n	12/11/1
Age at accident, mean (mean-max)	2 years and 1 month (2 months to 7 years)	Age mother, mean (min-max)/father, mean (min-max)	31 years (21–39)/35 years (24–46)
Aetiology, (n)	Scalds (9), contact (2) electricity (1)	Having one child/more children, n	8/16
Scars, n	6	Employed, n	24
Surgery, n	2	Single parent, n	2
Amputation	2	Norwegian/European, n	18/6

For this second interview, described in this article, the same parents were asked by the first author, whom had conducted the first interviews, to be interviewed again. One mother withdrew from the study, as she experienced the burn accident and treatment as very traumatic and emotional, and did not have the mental recourses to participate again. Three spouses were added (one mother and two fathers), who for different reasons had stayed at home and only visited the burn centre. They all felt that they had valuable experiences to share and wanted to participate. In total, 24 parents of 12 burn injured children participated (for 11 of the children both the mother and father participated). The interviews were conducted around the time of their three-month follow-up appointment at the burn centre’s outpatients’ clinic (June 2017 to November 2018; the number of days since the accident was on average 130 days (74 to 195 days)). Parents would be referred to a department psychologist, if the interview affected them too much. Some parents were offered this extra counselling, but all refused as they were already seeing either their general practitioner or a psychologist in their home town.

### 2.2. Setting

All participating parents had been hospitalized together with or visited their child at the same Norwegian burn centre. The burn centre can treat eight burn-injured patients simultaneously (both adults and children with their parents), of whom five can be intensive care patients. About 50 children are treated by a multi-disciplinary burn team each year. In Norway it was established by law in 2000, that one parent can stay at the hospital together with his/her child and this law applies to both parents in the cases of serious or life-threatening diseases (Helse- og omsorgsdepartementet [Ministry of health and care services] 2000). At discharge, the child and its parents receive an appointment at the outpatient clinic three months later. The burn team will then decide the child’s needs for further follow-up (involving half of the children of this study), e.g., scar management and follow-up after amputations.

### 2.3. Data collection

All 18 face-to-face interviews (Table II) were performed, audio-recorded and transcribed verbatim by the first author, a female Ph.D. student and former burn care nurse. By the preference of the parents, the interviews took place all over the country, in a location familiar to the parents. The parents were interviewed together or separately, by their own choice. An introductory question was used for all participants “Now it’s X months since we met, and I am excited to hear what has happened since the last time we met”. The parents told their overall reflections on their life situation today, and what had happened since the first interview. After a detailed summary of the first interview, made by the first author, a conversation followed about vivid memories from the hospitalization and wishes for improvements. Lastly, they were asked if they wanted to say more and how participating in this second interview had been. Parents participating for the first time were asked how their experience had been at home when their child was hospitalized andwhen the child had been discharge, their overall refletionc on their life situation today and how it had been to participate in this interview.

**Table II.** Characteristics of the interviews.

Interviews, n	18
Length of interview: Minutes, mean (min-max)	66 (38–155)
Total interview time: Hours, n	20
Interviewed together/alone, n	12/12
Place of interview, (n)	Burn centre (5), local hospital (2), home (10), café (1)
Presence of non-participants:	Sometimes the burn-injured child (<2 years old) of the parent(s) would be present during the interview.

### 2.4. Data analysis

All authors read the transcripts. The analysis was done by the first and last author, and commented on by the remaining two authors once during the end of the process to ensure internal validation and to verify that the analysis gave meaning to what the parents had said. A phenomenological-hermeneutic method inspired by the French philosopher Paul Ricoeur (P. S. Dreyer & Pedersen, 2009) was used to interpret the transcripts. Ricoeur claimed that a text, e.g., a transcript, holds “the others mind’s experiences” (Ricoeur, 1976, p. 73). To understand the meaning of the text, which he called “the sense of the text” (Ricoeur, 1976, p. 87), one has to use interpretation. Through a textual analysis, the significant things of the participants’ narration are discovered by the use of interpretation (P. Dreyer, 2019), in this case interpretation of the parents’ statements. Dreyer and Pedersen developed a method based on Ricoeur’s philosophy (P. S. Dreyer & Pedersen, 2009) and according to this Ricoeur- inspired method, a three-fold interpretation ends up with one or more themes, each with an explanatory text containing direct quotes. The software programme NVivo 12 Plus (QSR International Pty Ltd, 2021) was used to manage data during analysis. The Ricoeur-inspired approach involved three levels of analysis (Table III). First, all 18 transcripts were read as a whole and a short narrative text was created based on all 18 transcripts (Table IV). This first level is a naïve reading; it refers to the first impression obtained when reading the texts. After the naïve reading, all transcripts were read again. Level two is a structural analysis (Table V) consisting of three steps. First paragraphs from the transcripts were grouped into “meaning-bearing units” where one is looking for “what is said in the text” (citations). Secondly, we looked at the sentences in each coded group to understand “what does the text talk about”. In this phase, significance-bearing units were found, and a narrative text was created supported by the citations from the first step. Lastly, these narrative texts were given theme headings (third step), which illuminated the meaning of the text. These three steps are later presented in their entirety as the study’s results. The analytical process is like the conical pendulum that moves in a circle; one starts with the naïve reading, goes through all steps of the structural analysis and the transcripts, and goes back and forth between each of the steps. The third and final level in the method is a critical analysis and discussion. This step was done by all authors and during this process the theoretical perspective of lifeworld-lead healthcare (Dahlberg et al., 2009) was found suitable for the discussion and its five existential dimensions has therefore been used to structure the discussion. This illustrated how the method helps the researcher move from a surface interpretation to a depth interpretation. The third and final analysis level is presented in the discussion section of this article.

**Table III.** Three levels of analysis, the Ricoeur inspired method (P. S. Dreyer & Pedersen, 2009).

Level 1: Naïve reading		
The first overall reading written as a narrative text		
Level 2: Structural analysis:		
*First step:* *Meaning-bearing units* *– What is said in the text?* *(Citations)*	*Second step:* *Significance-bearing units* *– What does the text talk about?* *(Narrative text supported by citations from first step)*	*Third step: Themes* *(illuminating the meaning of the text)*
Level 3: Critical analysis and discussion		
The themes from the structural analysis are discussed in the context of relevant research literature and theory

**Table IV.** Naïve reading.

After their child’s hospital treatment of a burn injury, both child and parents were discharged from the burn centre. Shortly thereafter, they were back at the outpatient clinic for a three-month follow-up appointment. Around this time, most families were living their normal everyday life again. All parents focused on their child and the positive development of the wounds, scars or movement in different body parts, but stressed that they would never forget what happened. Shortly after coming home, almost all parents were thinking about the accident, but as time passed by, they thought less frequently about it. To some, though, visible disfigurements in their child was a daily reminder of the burn accident. Some parents were so affected by the burn accident and the stay at the burn centre that they were on sick leave, talking to their general practitioner or a psychologist to process their experience and feelings. Some had involved a lawyer or the patient safety authority to start an investigation regarding the accident. Many parents were uncertain about or struggled with different practicalities and economic issues. Some parents experienced that their child had not changed compared to prior to the accident, while others had noticed a change in their child’s behaviour, e.g., that the child was extra attentive or afraid of warm objects, sensitive to specific noises and woke up with nightmares. Some parents had concerns regarding potential future bullying from other children.

**Table V.** Example from the structural analysis.

*First step*: *Meaning-bearing units* *• What is said in the text?*	*Second step*: *Significance-bearing units* *• What does the text talk about?*	*Third step: Themes*
“No. It’s not forgotten, but it’s incorporated, I think” (Father no. 13). “But it’s a thing that you never forget, unquestionably” (Father no. 4). “It is definitively not something that I will ever forget” (Mother no. 12). “It’s a thing (red. feelings) that never disappears” (Father no. 24). “Yes. It’s within” (Mother no. 1). “But it’s still within” (Mother no. 12). “I think the hard thing is like, ehmm, those feelings. Because well, I think it’s still inside somewhere. This feeling from the accident. From that time” (Mother no. 19). “But you don’t go around and think about it on a daily basis, you don’t in a way. I try to, at least. Because now it is … , now he must live as normal, so then the focus is on that” (Father no. 14). “Of course, you have that feeling inside of you, the feeling from when it happened. […] it’s not always with me. It comes once in a while. Yes.” (Father no. 8). … (there are more citations)	The feelings that the parents had experienced at the time of the accident were so powerful that they had left a permanent impression. The feelings had become embodied. The parents would never forget this traumatic experience, and the feelings were now inside them. Even though the feelings were not present all the time, they were still inside the parents. Some things could trigger the parents’ memory and immediately make them remember what had happened. Different sounds, noises or a touch would bring them right back to the time of the accident. Their body reacted on its own. A father experienced to start crying when he heard the sound of a helicopter as it reminded him of the accident and the acuteness of the situation when a helicopter had landed on his front yard. Some immediately felt pain in their body and relived the stress and chaos when they heard a special sound. Another trigger mechanism was seeing and touching the scar on their child. …	Embodied feelings that will stay forever

### 2.5. Ethical consideration

This study follows the Declaration of Helsinki (World Medical Association, 1964/2013) and obtained ethical approval from the Norwegian Regional Committees for Medical and Health Research Ethics (REC, 2023), project number: 2017/54/REK.

The parents were ensured that their choice of participation did not impact their child’s medical care. Written and oral informed consent was obtained from all participants, and they could withdraw from the study at any time. The audio-records were kept at the university hospital’s secured research server and all transcripts were anonymized.

The first author was aware of the ethical challenges linked to conducting qualitative research, such as a asymmetric power, establishing confidentiality and trust, and establishing a relationship without it becoming too private (Haahr et al., 2014) and therefore the burn centre psychologists were on stand-by if needed. The mother who withdrew from the study, declined the offer to speak with a psychologist, but later told that everything had turned out well. The identities of participating parents were known to the first author only. The Consolidated criteria for reporting qualitative research (COREQ) was used to ensure full documentation of the study (Tong et al., 2007).

## 3. Results

Four themes will now present the in-depth understanding of parents’ lived experience of living with and caring for their burn-injured child in a home setting. Citations of mothers (M) and fathers (F) will be presented in the text.

### 3.1. Embodied feelings that will stay forever

The feelings that the parents had experienced at the time of the accident were so powerful that they had left a permanent impression. The feelings had become embodied. “No. It’s not forgotten, but it’s incorporated, I think” (F3). The feelings were not present all the time, however, the parents said they would never forget this traumatic experience “But it’s a thing that you never forget” (F4), and the feelings were now inside them. “Of course, you have that feeling inside of you, the feeling from when it happened. […] it’s not always with me. It comes once in a while” (F8).

Some things could trigger the parents’ memory and immediately make them remember what had happened. Different sounds, noises or a touch would bring them right back to the time of the accident. Their body reacted on its own. “So sometimes if I stand outside and hear a helicopter coming. It’s like that; tears are running. Yes. It all comes up, right” (F20). Some immediately felt pain in their body and relived the stress and chaos when they heard a special sound. “I feel it deep down in my stomach. Well, I feel pain in my stomach, and I start to think about everything that happened”. (M10). Another trigger mechanism was seeing and touching the scar on their child. Some felt hit and felt an anger inside themselves when they saw the scars. “I feel such a feeling that (pause) it hit me when I see my daughter in the pool and I see the scars” (F25). When their fingers touched the child’s scars applying cream to soften the scar tissue, they were reminded about the guilt they felt within themselves. These feelings of guilt were inside them and they had accepted that the feelings might become less troublesome but not disappear, “it’s something I will struggle with for the rest of my life. The guilt will never let go” (M12). Some even felt shame and did not share their feelings, “I actually didn’t say that to my general practitioner because I probably feel some shame” (M23). The reactions after the burn accident and hospitalization made some parents physically and mentally exhausted. They had strong bodily reactions in terms of being dizzy, having a high pulse, neck pain, headache, superficial breathing, tics, a tense and tired body, and they had to go on sick leave. The parent’s bodily reactions were embodied and were manifested into different physical symptoms.

### 3.2. Discharged to continue treatment at home without necessary skills

Many parents felt that before being discharged, they had not acquired the skills required to carry out the prescribed treatment at home. “And I was trying to see what they were doing. How to apply, how to bandage, and so on. And I was given different things to bring back home with me that I had to use at home … and then I was only told what to do” (M17). Most parents were told to go home the same day they were being discharged, and experienced this as very chaotic, “[I was told on] the same day. We had originally been told to stay for one more week” (M1). It was stressful to leave so abruptly as they had to perform the wound treatment, often for the first time. Moreover, they received all the information needed to continue treatment at home, while having at the same time to pack their things, contact the other parent, arrange for transportation and remember all the papers they had been given.

At home, all parents had to treat the child, which involved cream, wound treatment, tape or pressure garments. Many parents struggled as they felt that they had no qualification to complete the task. After their return home, some children started using pressure garments, which they received by currier post, but the parents gave up trying to get it on the child, because they did not know how to use it. “[…] we had these special clothes but we didn’t use it; we used normal bandage still because we couldn’t put that one on” (M19). In the end, they all needed help in applying the garments. Some parents were also left to themselves to do the wound treatment without morphine or helping nurses. “And she refused, she held on to herself, she curled up so that I had to open her body with force. I was the ugliest most cruel of them all. She screamed and shouted and … there was no dear mother, no way could I do the treatment on her without going to war with her” (M17).

Not having the proper training or the rights skills affected the parents and, for instance, made them feel insecure, ignorant, uninformed, forgotten, tired, exhausted, sweaty, crazy and sad.

### 3.3. Grieving over the lost past and fearing the unknown future

Some parents felt that they had destroyed their child by not having prevented the burn accident from happening, as the child had been fine with a perfect skin before the accident. “He was so fine before, and then we go and burn him” (M12). Some parents grieved over what they had lost. That their child had forever changed. “[…] when I, ehh, look at the picture from before this accident. And, I saw my son with a T-shirt and normal skin. I cried a little bit. But this is only for me, and this is for, not all the time, not every day, but sometimes” (M19). To some, it was a life-changing event, which divided their life in two “There is a before and an after” (M12) and a fact that their child would never be the same again. Most parents could not help noticing some physical, psychological and/or social changes in their child that affected their everyday life. The children showed altered sleeping habits, could start screaming without a clear reason, be shy towards or afraid of other people, and be very aware or scared of hot objects such as a stove, an oven or warm things. Nevertheless, all parents tried to focus on how well the healing of the wound turned out no matter how severe the injury had been. Still, the parents ruminated over their concerns and fears for the future. Some mothers were concerned about what aftereffects the child might have from the burn injury, the psychological stress and all the medicine received. The parents wondered if their child would get a scar. Fathers who had a daughter were particularly scared of how the girl would react. “I’m thinking a little about; what she will think herself about it when she gets older, this is what I think about. […] she’s a fine girl. I think, girls are, appearance means a lot” (F4).

### 3.4. Longing to reunite with familiar staff members

All parents had been looking forward to and expected to meet familiar staff members at the outpatient clinic, but were surprised and disappointed to meet new staff members. Some parents found the new staff members professional, caring and just as good as the one from the burn centre. Other parents had the opposite experience and felt that the new staff members were insecure and could not give good and satisfying answers, “they had no idea […] you don’t get an answer to your questions” (M14). The parents explained that while hospitalized, they had established a relationship with the staff members who had treated them. “[…] you met the same people in there, it was a bit … you felt that they knew my son. And we had a kind of a relationship with them” (F9). Seeing the same persons gave the parents a good feeling of trust, reassurance, comfort and made things go smooth. “[…] she kind of knew our boy and we felt we knew her and she knew us” (M14). The parents needed to meet someone who knew about the course of treatment, who had seen the burn injury before and could answer their questions.

A few parents went to the burn centre on their own initiative after their appointment at the outpatient clinic. They felt extremely happy, relieved and emotional to meet and thank the persons who had meant so much to them during the difficult time after the burn injury, “I gave her a hug and I told her how much she actually meant” (M17). For them, the reunion was a closure and almost therapeutic.

Many parents had also called the burn centre after returning home because they wondered about different things. They were happy that their questions were answered, but they wished that they could have talked to someone who knew them and their entire situation. All parents also wished for the burn centre to call them one or two weeks after discharged, because they did not want to disturb the busy staff members. “It’s really difficult for me to call them. […] it would have been very nice to get a phone call from them” (F8). Such a phone call would be good and make them feel less insecure and safer, seen and taken care of.

## 4. Discussion

The main finding of this current study was that parents were bodily affected by the burn injury, missed training and education prior to discharge to maintain treatment at home, grieved over what was lost and feared the future and, lastly, they longed to meet with familiar staff members. Since this is a follow-up study where we focused on the time after discharge, the findings shed a new and extended light on the entire illness trajectory. It seems obvious that the support and care parents received (or not received) during the hospital stay is significant for the time after discharge. Hence, we will discuss the findings using a theoretical perspective inspired by lifeworld-led care.

All parents were pleased with the medical treatment given at the burn centre (Lernevall et al., 2021). However, when they returned home, they missed having their caring activities supervised by professionals who could advise them about the long-term physical and emotional consequences of the burn injury. In this context, using the principles of lifeworld-lead healthcare may be helpful. Proponents of lifeworld-lead healthcare argue that in order for a patient to feel met, the carer needs to listen to their story, open up to their lifeworld, be touched and touch (Dahlberg et al., 2009). These are some of the needs expressed by parents in the present study. Lifeworld-lead healthcare gives patients and families a voice, involves them and provides a pivotal existentialistic care including “the existential dimensions of temporality, spatiality, embodiment, intersubjectivity and mood” (Dahlberg et al., 2009, p. 266). These dimensions are mirrored in our results and the discussion below will follow the structure of the five existential dimensions.

The experience from the burn accident affected the parents’ experience of time. Things could trigger them and bring them right back to the accident. This is exactly what the dimension of temporality’ is about. This, “temporality” is more than just the clock-time; it refers to the human experience of living with time, where for instance “The past can come up close, and the future recede” (Todres et al., 2007, p. 56). Many parents were concerned about their child’s scars and if they would affect the child in the future. In research studies, parental concern for the scars effect on the future is still a present problem and especially fathers with daughters feared how the girl would react (Andrews et al., 2018; McGarry et al., 2015; Ravindran et al., 2013b; Simons et al., 2015). One study reported significantly greater challenges with staring and teasing of children with visible scars (Rimmer et al., 2015). Another study examining bullying among burn-surviving children found that 61% of the children experienced being bullied at school (Rimmer et al., 2007). The parents’ concerns are therefore justified and should be approached and dealt with by the healthcare professionals.

When returning home, the parents needed to fit the treatment into their everyday lives. We may refer to the surrounding world, the world in which the parents live, using the concept of “spatiality”, i.e., “the environing world” (Todres et al., 2007, p. 56). When the parents leave the burn centre to go home, the perspective shifts. The role they had at the hospital is different at home. In other words, their role is dependent on where they are. At home, parents had to assume a new role of therapeutic caregiver responsible for their child’s treatment. This created a conflict between the roles of being a caring parent and a therapeutic caregiver at the same time. This conflict has been described previously (Andrews et al., 2018). For some parents, the treatment seemed to take all their focus and energy. The parents might be blinded by the treatment and forget everything around them. It is therefore important that parents receive the right support in order to help them focus on all aspect of their life and not only the treatment. The need for information or reassurance might be accommodated by offering easy access to information. In one study, a “burn-specific, parent-focused, peer-informed, supportive website designed to provide easy access to information and psychoeducation” (Heath et al., 2019) was tested and found to be highly acceptable to parents.

Our results show that feelings from the accident and hospital time had become incorporated within the parents. This process refers to the existential dimension of “embodiment”, which is the lived body (Todres et al., 2007). Hence, the body is more than simply a biological body; it is also a lived body with personhood (Toombs, 1988). A past threat can be embodied, and memories and concerns can “pervade the present […] or [be] projected into the future” (Toombs, 1988, p. 213). Sometimes, “The body is experienced as essentially out of the control of the self” (Toombs, 1988, p. 219). Some of the parents experienced this when the body acted on its own. A recent study found that 31% of mothers of burn-injured children had depressive symptoms, and 49% had symptoms of posttraumatic stress within the first month after the injury (M. R. Egberts et al., 2020). These percentages had decreased to 7% and 18%, respectively, 18 months after the burn injury, indicating that emotions in relation to the accident can affect parents in the long term. Our results highlight the need to remember that we have a living body that incorporates feelings. Therefore, these feelings should be addressed to prevent them from developing into a more permanent state.

“Intersubjectivity” means that we are in the world together with others (Todres et al., 2007). We see the phenomenon of “Intersubjectivity” in the parents longing to meet or be contacted by the staff members who had treated them. For some, being able to meet and thank them was almost therapeutic. Such a meeting may mean more to a parent than to a staff member who meets many parents. The parents whished that the burn centre would call them after they had returned home, and other parents who were contacted at home describe this as beneficial (Heath et al., 2018).

“Intersubjectivity” also emerges within the present study in the parents voicing a need to be properly educated by the healthcare professionals prior to discharge. Their experience of chaos and stress when leaving the burn centre indicates that the burn centre did not succeed in preparing them properly. The parents felt alone in being responsible for the after-treatment and felt that they lacked the necessary training or skills. Other studies also report parental concerns and challenges in providing the right after-care for their child (Andrews et al., 2018; Coy et al., 2019; McGarry et al., 2015). The parents in our study had all seen the wound procedure but not done it themselves. A way of strengthening parents’ skills and belief in themselves prior to discharge could be to ensure a higher level of parental involvement and let them participate in wound treatment procedures and execute the procedures themselves.

Parents who had been present during wound care procedures were more positive towards the future and were found to regain some control, and they preferred to be present even though it could be emotionally hard (M. R. Egberts, de Jong, et al., 2018). In one study, the families received outreach nursing input, which they experienced as supportive and helpful (Coy et al., 2019). Even so, the burn centre should offer more training prior to discharge.

Some parents grieved over their loss of a child with a perfect intact skin and some could feel the guilt of having caused this change inside themselves. The last of the existential dimensions is “mood” or “emotional attunement”. This dimension reminds us that we go through life and that our “lived experience is coloured by mood […] In sadness, other times and spaces may be longed for” (Todres et al., 2007, p. 57). Loss, grief and sadness related to permanent change have been described elsewhere (M. R. Egberts et al., 2019). In the present study, the parents all tried to orientate themselves in their new everyday life, but were repeatedly reminded of the accident and some parents might be locked in a grieving process. Grieving is described as a movement between two poles of coping; a loss-orientated pole (dealing with and preoccupation with what has been lost) and a restoration-orientated pole (adjustment to substantial changes and mastering new tasks) (Stroebe & Schut, 1999). It is important to pay attention to how the parents cope with their grief and feelings of guilt.

It is important that the care is adopted to the dimensions in the parents’ lifeworld. Using lifeworld-lead healthcare, their existential needs would also be remembered and they might feel that all their needs were more fully met.

## 5. Strengths and limitations

A limitation to this study was the unknown TBSA percentage (total body surface area), as the burn severity might affect the parents’ experience. Another limitation was that the parents were interviewed only once after coming home, and the study could be strengthened with a longer follow-up time and to learn if their needs changed over time.

However, interviewing the same parents on two occasions, both at hospital and at home as follow-up, gave a more in-depth understanding of their situation during this transition, and is seen as a strength. It also contributed with variation in relation to parental experiences. The varied parental experiences contributed to a new understanding of parental needs for support after being discharged, which is a mark of satisfaction within qualitative research in relation to sample size (Malterud, 2012).

All parents who participated in the second interview said that the interview had been meaningful and therapeutic. Our study hence offers an important contribution to knowledge in a field where “very little is known about family dynamics and experiences of fathers” (Bakker, Maertens, et al., 2013, p. 369). A suggestion for future research is to improve our knowledge on fathers. The qualitative guideline (COREQ) (Tong et al., 2007) was followed and strengthened the reporting of this study. One author (PD) has developed the Ricoeur-inspired method used. Her participation contributed with expert knowledge and aided the analysis process to a deeper level. Another strength of the present study is that all authors took part in the interpretation and presentation of the results.

## 6. Practice implications

This study offers a broadened understanding of parents’ experiences of caring for their burn injured child at home. First, the parents’ body is connected to the self and the surrounding world, and parents therefore need help in addressing their feelings to prevent them from evolving into more permanent negative feelings. Second, parents need to be given enough education and training to be able to continue the treatment at home with confidence. Burn centres ought to look at their discharge procedures to ensure this. Third, the parents’ experience of time can be affected by the burn injury, and their fear of potential future problems should be addressed. When supporting the parents, healthcare professionals should adjust their support according to each parent’s grieving process. Fourth, parents wanted to meet the same burn staff members who had treated them. Burn centres should consider the possibility of planning for staff members familiar with the parent to attend a follow-up meeting to give the parents an opportunity to make a closure. An essential point worth mentioning and exploring further is that parents may welcome follow-up initiatives from the healthcare staff when the parents are at home.

## 7. Conclusion

Based on 18 face-to-face interviews with 24 parents of burn-injured children, this study provides an in-depth understanding of the lived experience of parents of burn-injured children when caring for their child at home after discharge.

The analysis showed that the burn accident affected the parents’ everyday life in various ways. The feelings caused by the burn accident had been embodied within them and affected them. Parents were discharged to their home to continue the burn treatment without having the necessary skills. They grieved over what they had lost and feared the unknown future such as the aftereffects and scar development. Lastly the parents longed to reunite with familiar staff members when returning to the outpatient clinic.

It is important that healthcare professionals see returning home as part of the course of illness and that many of the challenges that parents face after returning home can be prevented by the right support during the hospital stay. It is therefore particularly important to be aware of the parents’ existential needs. The approach of lifeworld-lead healthcare could help health professionals provide comprehensive support to parents embracing the existential dimensions of temporality, spatiality, embodiment, intersubjectivity and mood.
